# A 7-year surveillance of the drug resistance in *Klebsiella pneumoniae* from a primary health care center

**DOI:** 10.1186/s12941-019-0335-8

**Published:** 2019-11-09

**Authors:** Guogang Li, Sheng Zhao, Sipei Wang, Yingqian Sun, Yangxiao Zhou, Xinling Pan

**Affiliations:** 1 Department of Clinical Laboratory, Affiliated Dongyang Hospital of Wenzhou Medical University, Dongyang, Zhejiang China; 2 Department of Biomedical Sciences Laboratory, Affiliated Dongyang Hospital of Wenzhou Medical University, No. 60 Wuningxi Road, Dongyang, Zhejiang China

**Keywords:** *Klebsiella pneumoniae*, Drug resistance, Urine, ESBL

## Abstract

**Background:**

The increased prevalence of *Klebsiella pneumoniae* infections and resistance rates are a current cause for concern. However, data for resistance rates in *K. pneumoniae* strains from primary hospitals and the resistance distribution among the different isolate sample sources are scarce.

**Methods:**

All the *K. pneumoniae* strains were isolated from patients who visited a primary health care center located in Central Zhejiang Province from January 2011 to December 2017. The specimens included blood, sputum, cervical secretions and urine. The species were identified by the Vitek 2 Compact Bacterial Identification and Monitoring System or VITEK-MS and the extended spectrum β-lactamase (ESBL) and drug resistance profiles were identified using the AST-GN13 Gram negative susceptibility card (VITEK-2). The genotype of strains from urine sources was analyzed by detecting TEM and SHV genes. Finally, the drug resistance rates among the isolates from different sample sources were analyzed using the Chi square test with SPSS software.

**Results:**

A total of 5319 *K. pneumoniae* strains were isolated in this study. Among the 20 antimicrobial drugs studied, the resistance rates of *K. pneumoniae* strains varied from 1.4% (ertapenem) to 23.1% (nitrofurantoin). The antibiotic resistance rates varied significantly among the isolate samples sources for all, with the highest rates for all antibiotics except for nitrofurantoin found in urine samples. In addition, the ESBL-positive rate in urine samples was 27.1%, significantly higher than that of cervical secretions (20.2%), blood (16.5%) and sputum (15.2%). Compared to the ESBL-negative strains, higher resistance rates were detected in the ESBL-positive strains. The most common genotype of isolates from urine was SHV (28%, 23/82), following by TEM (14.6%, 12/82).

**Conclusion:**

The highest resistance rates of *K. pneumoniae* strains to most antibiotics found in urine samples are partly due to the ESBLs, indicating that a special attention should be paid in the treatment of urinary tract infection.

## Background

As a common opportunistic pathogen, *Klebsiella pneumonia* (*K. pneumoniae*) causes a wide range of infectious diseases including, urinary tract and soft tissue infections, bacteremia, and pneumonia. It is also an important causative agent of serious community-onset infections, including pyogenic liver abscess and necrotizing pneumonia [1, 2].

As a result of the abuse and misuse of antimicrobial drugs, resistance to these agents is emerging as a worldwide crisis in the treatment of *K. pneumoniae*-associated infections [3]. A study of 3,132,354 antimicrobial susceptibility results from the United State revealed significantly increased drug resistance rates for all agents except tetracycline during the period from 1998 to 2010 [4]. Moreover, the increasing resistance of clinical isolates to the carbapenems (imipenem) and the fourth-generation cephalosporin cefepime, which are traditionally considered to be most effective agents against *K. pneumoniae*, is a major cause for concern [4, 5]. Clonal expansion of drug-resistant strains has resulted in outbreaks in hospitals in several countries [6], leading to a high mortality in infected individuals.

The exact mechanism underlying drug resistance in *K. pneumoniae* strains remains to be clarified, although the extended-spectrum β-lactamases (ESBL) have been reported to play an important role [7]. ESBLs in *K. pneumoniae* often confer resistance to advanced generation cephalosporins, leading to the therapeutic failure of these agents. In addition, ESBL-associated plasmids often carry genes encoding co-resistance to other antibiotics, resulting in multi-drug resistance phenotypes [8].

Investigations of the prevalence of drug-resistant strains in clinical hospitals are urgently required to determine optimal treatment strategies in patients with *K. pneumoniae* infections. However, most of the data about the prevalence and drug resistance of *K. pneumoniae* in China were obtained in large city hospitals, where the patients are more likely to have severe infections [9, 10]. Moreover, most of the studies focused on the strains causing infection in a specific cohort [11, 12] (such as infants, the elderly or intensive care unit (ICU) patients) or infection site (such as blood and liver) [13, 14], while others have investigated the molecular epidemiology of the hypervirulent strains [15].

However, the global distribution and drug resistance of *K. pneumoniae* strains among the clinical samples and the contribution of ESBL to drug resistance remain to be elucidated. Therefore, we conducted a retrospective analysis of the *K. pneumoniae* strains isolated in a primary hospital located in southeastern China during the period from 2011 to 2017.

## Materials and methods

### Clinical isolate collection

The strains were isolated from patients (outpatients and inpatients) who visited the Affiliated Dongyang Hospital of Wenzhou Medical University from January 2011 to December 2017. The patients were suspected of infection according to the clinical symptoms when they visited the hospital. The samples sources were collected dependent on the suspected tissues of infection including blood, urine, sputum, cervical secretions, pus and others, but not feces. Repeated samples identified in the reviewing process were excluded from our analysis. All the clinical data was retrospectively reviewed anonymously, and the study was approved by the Ethics Committee of Dongyang People’s Hospital Ethics Committee and Institutional Review Board.

### Species identification and antimicrobial susceptibility test

The clinical samples were directly smeared on the Columbia Blood Agar Base (BIO KONT, Wenzhou, China. http://www.bio-kont.cn/) and then incubated at 37 °C for 18–24 h. When the colonies formed and the suspected *K. pneumoniae* colonies were collected based on the morphology. The species were identified in a clinical laboratory using a Vitek 2 Compact Bacterial Identification and Monitoring System (Biomerieux, France) or a mass spectrometry microbial identification system (Biomerieux, France). Susceptibility to antimicrobial agents commonly used to treat infections was analyzed using the AST-GN13 or AST-N334 (VITEK 2) based on the Clinical and Laboratory Standards Institute (CLSI) breakpoints [16]. The ESBL-positive phenotype was determined using VITEK 2 according to the manufacturer’s instructions [17]. The resistance of *K. pneumoniae* to imipenem was further confirmed by the minimum inhibitory concentration test (resistant phenotype: ≥ 1.0 μg/mL). The reference strains *Escherichia coli* ATCC 25922, *Streptococcus pneumoniae* ATCC 49619, *Staphylococcus aureus* ATCC 29213 *Staphylococcus saprophyticus* ATCC BAA750 and *K. pneumonia* ATCC 700324 were used as controls.

### The ESBL gene detection of isolates from urine source

A total of 82 *K. pneumoniae* isolates were from urine sample from those patients who visited the health care center in 2017. The strains were recovered from − 80 °C refrigerator and cultured on the Columbia blood agar plates. The colonies formed after an incubation of 24 h at 37 °C. Then several clones were collected and resuspended in 50 μL deionized water. The DNA was extracted from the microorganism by being boiled at 100 °C for 7 min. After a centrifugation of 10,000 rpm for 5 min, the supernatant containing DNA was collected.

The primers for detecting the TEM and SHV genes were described previously (Additional file 1: Table S1) [18, 19]. Polymerase chain reaction was carried out in 20 μL volumes containing 1 μL DNA template, 0.2 mM of dNTPs, 0.25 μM of each primer, and 0.1 U of Taq polymerase (Takara, Japan) in 1× PCR buffer. Amplification of DNA was performed in a thermocycler (Biorad, USA). The cycling parameters were: pre-denaturation (95 C for 3 min), 35 thermal cycles of denaturation (95 °C for 30 s), annealing (55 C for 30 s), extension (72 C for 1 min), and final proliferation temperature (72 C for 5 min). All the PCR products were analyzed in 1% agarose gel containing gel staining dye (TRANGENE, China) in Tris-acetate-EDTA buffer, and the gel was photographed using gel documentation system (Bio-Rad, USA).

### Statistical analysis

The data were managed using WHONET 5.6 version. Differences between resistance rates were analyzed by Chi squared test using SPSS 20.0 version. *P*-values < 0.05 were considered to indicate statistical significance.

## Results

**Fig. 1:**
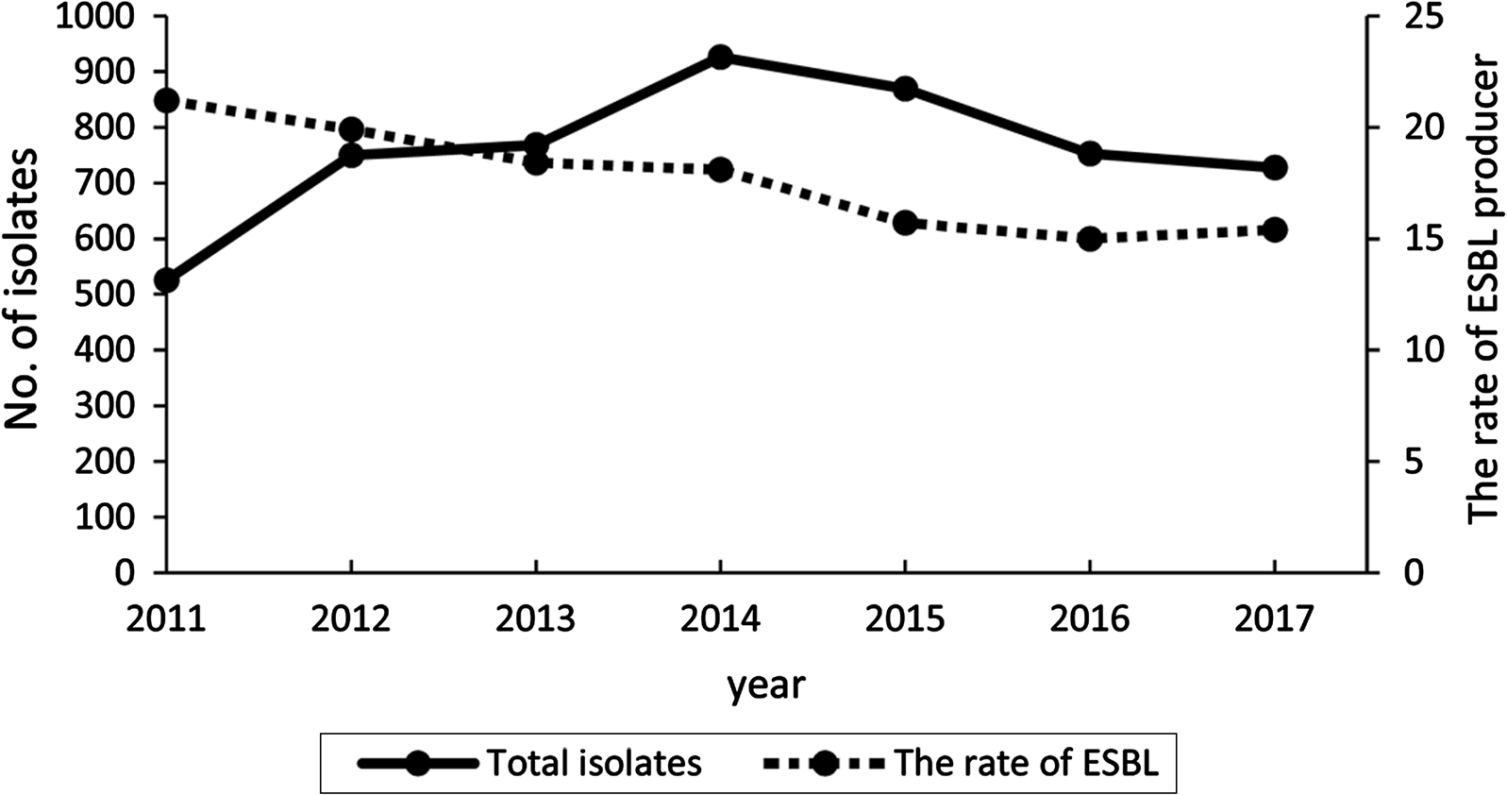
The total number and ESBL producer percentage of *Klebsiella pneumoniae* in clinical samples by year

A total of 5319 *K. pneumoniae* isolates were obtained from January 2011 to December 2017. The isolation rate of *K. pneumoniae* in clinical samples ranged from 10.3 to 11.4%, with very small differences by year (Fig. 1). However, the total number of *K. pneumoniae* isolated reached a peak in 2014 and then decreased. The main sample source of *K. pneumoniae* was sputum (3319, 62.4%), followed by cervical secretions (514, 9.7%), urine (495, 9.3%), blood (300, 5.6%), pus (294, 5.5%) and others (397, 7.5%).

As a known potential drug-resistant pathogen, *K. pneumoniae* has received increasing attention in clinical infections. Thus, in this study, we investigated the resistance of *K. pneumoniae* to commonly used antimicrobial drugs. The resistance rates varied for the different drugs, from 1.4% (ertapenem) to 23.1% (nitrofurantoin) (Table 1). Less than 10% of the isolates showed resistance to ceftazidime, levofloxacin, ciprofloxacin, cefepime, tobramycin, piperacillin/tazobactam, imipenem, amikacin, cefotetan and ertapenem. In contrast, 10% to 23% of *K. pneumoniae* strains showed resistance for nitrofurantoin, cefazolin, sulbactam/ampicillin, ceftriaxone, sulfamethoxazole/trimethoprim, aztreonam and gentamycin. From 2011 to 2016, the rates of resistance to ceftriaxone decreased from 25.6 to 18.5%. However, for ciprofloxacin, the resistance rates increased from 2011 to 2017. The remaining drugs showed only a slight fluctuation in their resistance rates throughout the period of the study.

**Table 1:** The drug resistance rates of *Klebsiella pneumoniae* by year

Antimicrobial drugs	No. (%)^a^	2011	2012	2013	2014	2015	2016	2017
NIT	5203 (23.1)	13.7	20.4	24.8	29.6	33.1	12.8	20.7
CZO	4476 (20.3)	25.6	21.6	20.3	20.4	18	18.5	–
SAM	5195 (20.6)	19.8	20.5	19.8	22.4	18.4	20	22.8
CRO	5204 (18.6)	23.7	21.1	18.6	19.5	15.6	17	17.2
SXT	5205 (17.3)	17.5	19.9	19.2	18	14.5	15.4	17.2
ATM	5202 (10.4)	10.4	11.4	10.1	11.8	9	9.3	10.6
GEN	5044 (10.4)	12.7	11.8	10.5	12.2	8.9	9.6	8.1
CAZ	5189 (7.5)	8.6	9.7	6.1	7.8	7.1	7	6.4
LVX	5202 (5.5)	3.9	7.5	5.8	6.1	3.7	5.2	5.7
CIP	2381 (7.0)	–	–	–	4.1	5.9	7.1	8.3
FEP	5201 (4.7)	4.4	5.3	3.7	5.5	3.4	4.5	6.4
TOB	5202 (3.9)	5.3	4.7	2.9	4.8	2.7	3.9	3.7
TZP	5189 (2.5)	2	2.7	2.1	3.4	1.5	2.4	3.3
IPM	5207 (1.8)	1.5	1.8	1.1	2.7	0.9	2	2.2
AMK	5201 (2.0)	3.5	2.8	1.2	2.3	0.9	1.6	2.1
CTT	5200 (2.0)	2.4	2.4	1.3	2.1	1.5	1.9	2.8
ETP	5141 (1.4)	1.8	3.4	1.8	2.9	0	0	0.1

– Data not available

*NIT* nitrofurantoin, *CZO* cefazolin, *SAM* sulbactam/ampicillin, *CRO* ceftriaxone, *SXT* sulfamethoxazole/trimethoprim, *ATM* aztreonam, *GEN* gentamycin, *CAZ* ceftazidime, *LVX* levofloxacin, *CIP* ciprofloxacin, *FEP* cefepime, *TOB* tobramycin, *TZP* piperacillin/tazobactam, *IPM* imipenem, *AMK* amikacin, *CTT* cefotetan, *ETP* ertapenem

^a^The total number of strains profiled with each given antibiotic

To investigate the potential difference in drug resistance between different sources of samples, we analyzed the *K. pneumoniae* isolated from urine, sputum, blood and cervical secretions. As shown in Table 2, significant differences in resistance rates to almost all the drugs were observed between the sample sources. Overall, the strains isolated from urine exhibited the highest resistance rates for all the drugs except nitrofurantoin. With regard to some antimicrobial drugs, such as sulfamethoxazole/trimethoprim and ceftazidime, the resistance rates in isolates from urine samples were more than two-fold greater than those isolated from sputum and cervical secretions. In addition, less than 1% of the *K. pneumoniae* strains isolated from the cervical secretions were resistant to piperacillin/tazobactam, imipenem, amikacin, cefotetan and ertapenem. The distribution of sample sources per year was displayed (Additional file 1: Table S2), with a slight fluctuation across the years.

**Table 2:** The antimicrobial drug resistance rates of *Klebsiella pneumoniae* from different resources

Antimicrobial drug	Blood, 300	Sputum, 3319	Genital secretion, 514	Urine, 495	*P* value
NIT	35.2	20.9	20.7	30.7	< 0.001
CZO	23.6	19.5	22.6	35.1	< 0.001
SAM	20.7	18	21.1	32.6	< 0.001
CRO	18.6	16.6	19.3	29.1	< 0.001
SXT	16	14	24	28.3	< 0.001
ATM	11.3	9.2	9.4	18.8	< 0.001
GEN	10.1	8.8	11.9	16.9	< 0.001
CAZ	9	6.5	5.9	14.2	< 0.001
LVX	7.6	3.6	4.7	14.8	< 0.001
CIP	6.1	5.3	8.4	14.5	< 0.001
FEP	5.5	4.2	1.8	10.7	< 0.001
TOB	3.2	3.3	2.7	8.8	< 0.001
TZP	3.2	2.5	0.2	4.7	< 0.001
IPM	2.9	2	0	3.6	< 0.001
AMK	2.3	1.7	0.2	4.7	< 0.001
CTT	1.7	2	0.2	4.3	< 0.001
ETP	0.9	1.5	0.2	2.7	< 0.001

*NIT* nitrofurantoin, *CZO* cefazolin, *SAM* sulbactam/ampicillin, *CRO* ceftriaxone, *SXT* sulfamethoxazole/trimethoprim, *ATM* aztreonam, *GEN* gentamycin, *CAZ* ceftazidime, *LVX* levofloxacin, *CIP* ciprofloxacin, *FEP* cefepime, *TOB* tobramycin, *TZP* piperacillin/tazobactam, *IPM* imipenem, *AMK* amikacin, *CTT* cefotetan, *ETP* ertapenem

To investigate the mechanism responsible for the higher resistance rates among *K. pneumoniae* strains isolated from urine samples, we compared the proportion of ESBL-positive strains isolated from the different sources. Overall, ESB-positive strains accounted for 18.7% (963/5161) of the strains available for ESBL-testing. As expected, the rate of ESBL-positive strains in urine were 27.1%, significantly higher than those in cervical secretions (20.2%, *P* = 0.009), blood (16.5%, *P* < 0.001) and sputum (15.2%, *P* < 0.001). We also analyzed the association between ESBL expression and drug resistance by comparing the resistance rates between the ESBL-positive and ESBL-negative strains (Table 3). Among the ESBL-negative strains, more than 90% were susceptible to all the antimicrobial drugs investigated, except nitrofurantoin. Compared to the ESBL-negative *K. pneumoniae* strains, the ESBL-positive strains showed higher resistance rates to all of antimicrobial drugs except imipenem and cefotetan. For example, less than 10% of the ESBL-negative strains showed resistance to cefazolin, sulfamethoxazole, ceftriaxone and sulbactam/ampicillin, whereas more than 60% of ESBL-positive strains showed resistance to these drugs.

**Table 3:** The drug resistance rates between ESBL (−) and ESBL (+) *Klebsiella pneumoniae*

Antimicrobial drugs	ESBL (−), n = 4198	ESBL (+), n = 963	*P* value
NIT	19.8	39.3	< 0.001
CZO	4.7	95.8	< 0.001
SAM	8	80.3	< 0.001
CRO	2.6	93.3	< 0.001
SXT	8.2	60.7	< 0.001
ATM	2.2	49.1	< 0.001
GEN	3.4	44.2	< 0.001
CAZ	2.5	30	< 0.001
LVX	2.7	17.9	< 0.001
CIP	2.7	29.3	< 0.001
FEP	1.7	19	< 0.001
TOB	1.8	13.4	< 0.001
TZP	2.2	3.8	0.005
IPM	1.9	1.6	0.491
AMK	1.4	4.4	< 0.001
CTT	1.8	2.1	0.554
ETP	1.3	2.6	0.005

*NIT* nitrofurantoin, *CZO* cefazolin, *SAM* sulbactam/ampicillin, *CRO* ceftriaxone, *SXT* sulfamethoxazole/trimethoprim, *ATM* aztreonam, *GEN* gentamycin, *CAZ* ceftazidime, *LVX* levofloxacin, *CIP* ciprofloxacin, *FEP* cefepime, *TOB* tobramycin, *TZP* piperacillin/tazobactam, *IPM* imipenem, *AMK* amikacin, *CTT* cefotetan, *ETP* ertapenem

The most common ESBL gene was SHV gene (28%, 23/82), following by TEM (14.6%, 12/82). The percentage of SHV genes in ESBL producer (14.3%, 3/21) was lower than that in non-ESBL producer (32.8%, 20/61). The percentages of TEM genes in ESBL producer (19.4%, 4/21) strains were higher than in non-ESBL producer (13.1%, 8/61) strains.

## Discussion

Along with a high prevalence, *K. pneumoniae* is a known pathogen antibiotic resistant pathogen. As expected, there was a high variability among the isolates investigated in the prevalence of resistance to the different antibiotics, which could be explained by distinct resistance mechanisms [3], including the expression of ESBLs in *K. pneumoniae* strains. The decreased number of isolates and the rate of ESBL producer in this study may be due to more acknowledge of the *K. pneumoniae* infection, which could be diagnosed by combination with other examination methods.

In general, the ESBL-positive strains were resistant to advanced generation cephalosporins [7], with resistance rates to ceftriaxone, ceftazidime and cefepime found to be more than tenfold higher than those of the ESBL-negative strains (Table 3). In addition, the ESBL-positive strains showed higher resistance rates to aminoglycosides (gentamycin, amikacin and tobramycin), fluoroquinolones (ciprofloxacin) and other agents (Table 3). The cross-resistance in ESBL-positive strains may be due to the plasmids carried by the pathogens, resulting in the emergence and spread of multi-drug resistant strains among the individuals [3, 7, 8]. Thus, investigations of the ESBL-positive strains are required to elucidate the mechanism underlying multi-drug resistance and to identify potential targets of new antibiotics.

We found that the resistances rates varied significantly among the *K. pneumoniae* strains isolated the different sample sources. The highest resistance rates to most of the drugs tested were identified in strains isolated from urine, which may be due to the highest rate of EBSL-positive strains isolated from this source. Indeed, there is a demographic variety of ESBL-positive rates in *K. pneumoniae* strain isolated from urine. At 27.1%, the rate of ESBL-positive strains in urine identified in our study is higher than that (18.4%) reported in Nepal [14], but lower than that (47.5%) in Saudi Arabia [15]. Although there was a significant increase in the nitrofurantoin resistance rates of ESBL-positive strains isolated during the study period, the highest resistance rates were detected among the strains isolated from blood, which exhibited a lower ESBL-positive rate. Thus, caution should be exercised in the use of nitrofurantoin to treat *K. pneumoniae* infection of the blood due to the possibility of resistance leading to increased mortality [20–22]. A survey conducted in the United States also revealed significant differences in drug resistance rates among isolates from different sample sources, with higher resistance rates in *K. pneumoniae* strains isolated from the lower respiratory tract than those from urine [4]. In contrast to our findings, the same study showed that the resistance rates to all the antibiotic agents were the lowest in the urine sample isolates.

Although the distribution of ESBL genes in strains from urine source could not explain the higher resistance, it could indicate that the ESBL genes may be served as molecular detection markers, instead of contributing to the ESBL phenotype [23]. In another way, the higher resistance of strains from the urine may be speculated by the mechanism in pathogenicity in urinary tract system as reported previously [24]. The isolates should overcome several obstacles including the flow of urine and low pH, and finally colonized in the urinary tract. This ability may result from unique component such as type 1 fimbriae, which was expressed in strains from urinary tract but not from gastrointestinal tract or lungs [25]. Furthermore, fimbriae expression would be of great importance in biofilm formation and the biofilm was a structure where the strains owned more resistance to chemicals [26].

Comparisons of the resistance rates of *K. pneumoniae* strains isolated from different sample sources are rare; therefore, a comprehensive survey of the potential differences among the strains from different sample sources is urgently required. Moreover, the differences in drug resistance among clinical samples from various sources indicates that optimal drug strategies should be considered based on the site of *K. pneumoniae* infection.

## Conclusions

In conclusion, our investigation of the resistance rates of *K. pneumoniae* strains from a primary hospital revealed that the resistance rates of strains in urine samples to most antibiotics are higher than those in blood, sputum and cervical secretion from females. This is associated with a corresponding increase in number of ESBL-positive strains. Therefore, the contribution of ESBL expression to antimicrobial resistance or cross-resistance warrants further investigation.

## Supplementary information

**Additional file 1.** Additional tables.

## Data Availability

All data generated or analyzed during this study are included in this published article.

## Abbreviation

ESBL: extended-spectrum β-lactamases
