# Supplementary material for: A 7-year surveillance of the drug resistance in Klebsiella pneumoniae from a primary health care center
Source: Ann Clin Microbiol Antimicrob. 2019 Nov 9;18:34. doi: 10.1186/s12941-019-0335-8 (PMC6842199; doi:10.1186/s12941-019-0335-8)
Supplement: Supplementary file 1 — Additional file 1. Additional tables. [file 12941_2019_335_MOESM1_ESM.docx]

Additional Table S1. The primers of ESBL genes involved in this study.

| ESBL genes | Primers | Sequences of primers | Product length |
| --- | --- | --- | --- |
| TEM | TEM-F | TCGGGGAAATGTGCG | 972 |
|  | TEM-R | TGCTTAATCAGTGAGGCACC |  |
| SHV | SHV-F | GCCTTTATCGGCCTTCACTCAAG | 898 |
|  | SHV-R | TTAGCGTTGCCAGTGCTCGATCA |  |

Additional Table S2. The distribution percentages of sample resources per year from 2011 to 2017.

| Sample resource | Distribution percentage (%) | | | | | | |
| --- | --- | --- | --- | --- | --- | --- | --- |
|  | 2011 | 2012 | 2013 | 2014 | 2015 | 2016 | 2017 |
| Urine | 7.4 | 8.4 | 12.6 | 9.1 | 8.6 | 9.3 | 12.0 |
| Genital secretion | 9.3 | 11.9 | 11.3 | 11.1 | 8.6 | 8.0 | 7.8 |
| Sputum | 69.7 | 70.7 | 66.4 | 67.7 | 66.1 | 62.3 | 56.7 |
| Blood | 4.6 | 4.0 | 4.2 | 3.9 | 7.0 | 7.9 | 9.3 |
| Others | 9.0 | 4.9 | 5.5 | 8.2 | 9.7 | 12.5 | 14.1 |

Additional Table S3. The primers in detecting ESBL genes of Klesbia pneumoniae from urine sample source.

| Genes |  |  |  |
| --- | --- | --- | --- |
| TEM |  |  |  |
|  |  |  |  |
| SHV |  |  |  |
|  |  |  |  |
| CTX-M1 |  |  |  |
|  |  |  |  |
| CTX-M2 |  |  |  |
|  |  |  |  |
| CTX-M8 |  |  |  |
|  |  |  |  |
| CTX-M9 |  |  |  |
|  |  |  |  |
